# Clinical profile and initial treatment of non-small cell lung cancer: a retrospective cohort study at the Uganda Cancer Institute

**DOI:** 10.4314/ahs.v21i4.30

**Published:** 2021-12

**Authors:** Solomon Kibudde, Bruce James Kirenga, Martin Nabwana, Fred Okuku, Victoria Walusansa, Jackson Orem

**Affiliations:** 1 Uganda Cancer Institute, Department of Medical Oncology; 2 College of Health Sciences, Makerere University; 3 Makerere University Lung Institute

**Keywords:** Non-small cell lung cancer, Uganda, erlotinib, lung cancer, Uganda Cancer Institute

## Abstract

**Introduction:**

Lung cancer is a major global public health burden constituting 11.6% of all new cancer diagnoses and 18.4% of all cancer-related mortality

**Purpose:**

To describe the clinical profile and initial treatment of non-small cell lung cancer in Uganda

**Methods:**

We reviewed charts of a cohort of patients with a histologically confirmed diagnosis of non-small cell lung cancer, treated between January 2013 and November 2015 at the Uganda Cancer Institute.

**Results:**

A total of 74 patients met the inclusion criteria. The median age was 56 years (IQR 47–70), with 16.2% below the age 45 years, and 51% were female. Only 10 percent were active smokers and the most frequent histological subtype was adenocarcinoma (71%). The majority (91.9%) had stage IV disease at diagnosis and frequent metastases to contralateral lung, liver, and bones. Twenty-seven (27) patients received platinum-based chemotherapy, while 27 patients received erlotinib, and only 4 patients received palliative thoracic radiotherapy. The median survival time was 12.4 months, and the overall response rate was 32.7%. There was no survival difference by type of systemic treatment, and on multivariate analysis, poor performance status was predictive of adverse outcomes (p < 0.001).

**Conclusions:**

Patients with non-small cell lung cancer in Uganda frequently presented with late-stage disease at diagnosis. The majority of patients were female, never-smokers, and had predominantly adenocarcinoma subtype.

## Introduction

Lung cancer is a major global public health burden constituting 11.6% of all new cancer diagnoses and 18.4% of all cancer-related mortality(1). Worldwide, lung cancer is the most commonly diagnosed cancer with 2.1 million new cases reported in 2018, and 1.8 million deaths attributed to lung cancer alone in the same year^1^. Lung cancer incidence and mortality varies globally, with a strong association with tobacco use reported in several works of literature^2^,^3^. Although prevention and screening interventions are continually broadcasted to the general public, the incidence of lung cancer continues to rise particularly among women^3^, even in low-smoking prevalence countries in sub-Saharan Africa^4^. The 5-year survival of lung cancer is unsatisfactory and varies depending on the region with 2% (Libya) and 30% (Japan)^3^.

Traditionally, lung cancer is divided into two major histological types namely small cell lung cancer (SCLC) and non-small cell lung cancer (NSCLC). Non-small cell lung cancer constitutes 80–85% of all lung cancer cases and represents a heterogeneous group of diseases with several histological subtypes including adenocarcinoma, squamous cell carcinoma, large cell carcinoma, and NSCLC not otherwise specified (NOS). The histological type determines the therapeutic approach. Whereas SCLC responds favourably to chemotherapy and radiotherapy, patients with NSCLC have a relatively poor response to traditional chemotherapy and hence further histological characterization is required to identify therapeutic targets for immunotherapy and targeted therapy. The presence of the epidermal growth factor receptor (EGFR) mutation is associated with a 70–80% response to first-line tyrosine kinase inhibitor (TKI) therapy^5^.

In Uganda, lung cancer incidence is on the rise. In 2018, GLOBOCAN estimated 486 new cases of lung cancer with 461 deaths anticipated in the same year(1); and yet the real incidence could be higher than reported due to limited availability of diagnostic equipment(6). To our knowledge, no prior publications were describing the clinical profile of lung cancer in Uganda; specifically, in the setting of ongoing constraints in access to cancer diagnosis, staging, and treatment interventions in the country. Therefore, the study aimed to describe the clinical profile and initial treatment of non-small cell lung cancer in patients seen at the Uganda Cancer Institute

## Methods and materials

### Study design and participants

We conducted a chart review to describe the clinical profile and initial treatment of non-small cell lung cancer at the Uganda Cancer Institute between January 2013 and November 2015. Patients were aged 18 years and above, with a histological diagnosis of non-small cell lung cancer. Records with gross missing information particularly regarding histology and treatment administration were excluded from this analysis. A total of 77 patients were identified during the study period of which 74 patients met the inclusion criteria.

### Treatment and patient evaluation

All patients were evaluated and treated at the lung cancer clinic at the Uganda Cancer Institute. At the first visit, the clinical evaluation pathway included; full history, complete physical examination, review of all staging investigations (disease stage was assigned using the 7^th^ edition of the TNM classification by the American Joint Committee on Cancer (AJCC)/ Union for International Cancer Control (UICC)), and therapeutic management. Treatment interventions were tailored to the patient's age, disease stage, comorbidities, performance status, patient preferences, and improving the quality of life. Molecular subtyping was not routinely available, and lung cancer multidisciplinary tumour boards were infrequent. Patients were offered either platinum-based chemotherapy, or thoracic radiotherapy, or targeted therapy with erlotinib, or best supportive care. Palliative care needs were assessed at diagnosis and referral to the palliative care team initiated for joint care.

### Procedures and treatment outcomes

Data were abstracted from medical records onto pre-tested case report forms. These data included; age, gender, performance status, histological type, biopsy accession type, symptoms and their duration, disease stage at diagnosis, sites of metastatic disease, and treatment modalities. A bronchus protocol computerized tomography (CT) scan was performed to evaluate the response and graded using the response evaluation criteria in solid tumours (RECIST v1.1).

### Statistical methods

Descriptive statistics were used to summarize the continuous variables in tables using mean, median, and range. Categorical variables were presented as proportions and percentages. We computed the overall survival as time interval from date of diagnosis to death from any cause. Cox proportional hazards model was used to test for predictors of survival. Response rates were reported as proportions and compared using the chisquare test. A p-value of less than 0.05 was regarded as significant. Data analysis was performed using the Stata 14.0 computer software

### Ethical considerations

The permission to conduct the study was sought through the office of the research and ethics committee at the Uganda Cancer Institute. The study was done following Good Clinical Practice guidelines.

## Results

During the study period, 74 patients met the inclusion criteria. The median age at presentation was 56 years (IQR 47 – 70 years); with 12 participants (16.2%) under the age 45 years and approximately 51% (38 patients) were female. The majority of patients were referred from private hospitals (33%), and equal proportions resided in two large districts in central Uganda, Kampala, and Wakiso districts each contributing 16% of patients with lung cancer. Cough and chest pain were the most frequent presenting symptoms contributing 54.2% and 26.4% respectively. Only ten percent (10%) of these patients were active smokers at the time of cancer diagnosis, 19% were ex-smokers and the majority (68.9%) were non-smokers. Eleven participants (14.9%) of the participants were HIV positive at the time of lung cancer diagnosis, and sixty-eight (91.9%) of the participants had stage IV lung cancer. The most frequent sites of metastases were contralateral lung, liver, and bones in that order, and the majority (54%) had at least two sites of metastatic non-small cell lung cancer (table 1).

**Table 1:** Baseline clinical and pathological characteristics of patients with lung cancer

Characteristic	Frequency (n=74)	Percentage%
**Sex**		
Male	36	48.7
Female	38	51.3
**Age (Years)**		
Median (IQR)	56 (47, 70)	
Below 45	12	16.2
≥45	62	83.8
**Disease-specific** **symptoms, *n=72***		
Shortness of breath	10	13.9
Cough	39	54.2
Chest pain	19	26.4
Other	4	5.6
**Performance status**		
1–2	41	55.4
3–4	12	16.2
Unknown	21	28.3
**Histological subtype, *n=69***		
Adenocarcinoma	49	71.0
Squamous cell carcinoma	9	13.0
Adenosquamous	1	1.5
Bronchoalveolar carcinoma	4	5.8
Adenocarcinoma, NOS	6	8.7
**Stage at diagnosis**		
III	6	8.1
IV	68	91.9
**Number of sites of metastases**		
1	23	46
2	20	40
3	7	14
**HIV status**		
Negative	46	62.2
Positive	11	14.9
Unknown	17	21.9
**Tobacco Use**		
Current smoker	7	9.5
Ex-smoker	14	18.9
Never smoker	51	68.9
Unknown	2	2.7
**EGFR mutation status**		
Positive	1	1.3
Negative	5	6.8
Unknown	68	91.9

Adenocarcinoma was the most frequent histological subtype on both histological and cytological specimens constituting 71% of patients. Squamous cell carcinoma was identified in 13.04% of patients, followed by bronchoalveolar carcinoma (5.8%), adenosquamous carcinoma (1.45%), and adenocarcinoma NOS (8.7%).

### Initial treatment

All study participants were treated with palliative intent using three main treatment modalities. Twenty-seven (36.5%) patients received platinum-based chemotherapy regimens, twenty-seven (36.5%) patients received targeted therapy with erlotinib; a tyrosine kinase inhibitor against EGFR mutation, four (5.4%) patients received palliative thoracic radiotherapy, and sixteen (21.6%) patients received best supportive care (BSC) which involved pain and other symptoms management by the palliative care specialist. Following initial treatment, only 10 of the 54 patients previously treated with either platinum-based chemotherapy or erlotinib were offered second-line treatment (Table 2).

**Table 2:** Initial treatment for non-small cell lung cancer at UCI

Treatment	n	Percentage
**Modalities**		
Platinum-based chemotherapy	27	36.5
Thoracic radiotherapy	4	5.4
Erlotinib	27	36.5
Best supportive care	16	21.6
**Chemotherapy (n=27, 36.5%)**		
Cisplatin/Paclitaxel	5	9.3
Carboplatin/Paclitaxel	16	29.6
Cisplatin/Pemetrexed	3	5.6
Cisplatin/Etoposide	2	3.7
Carboplatin/Docetaxel	1	1.9
**Second-line treatment (n=54)**		
Yes	10	18.5
No	43	79.6
Unknown	1	1.9

### Response to treatment

**Figure 1:**
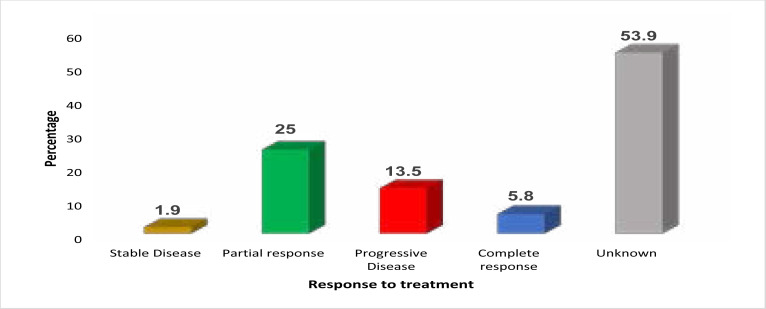
Bar graph showing response categories following initial treatment

Response evaluation with a computerized tomography scan was performed in 52 of 54 patients that received systemic therapy with either platinum-based chemotherapy or erlotinib. The overall response rate to initial treatment was 32.7% (95% CI: 21.2%–46.8%), and in seven (7) patients (13.5%), there was disease progression on first-line treatment with either chemotherapy or erlotinib (Figure 1).

### Survival

Overall, the median survival time was 12.4 months [IQR (3.0 <=>30.3 months)], (Figure 2A). In patients treated with platinum-based chemotherapy, the median survival time was 13.1 months [IQR (6.7–30.3 months)], as compared to patients treated with erlotinib who did not reach median survival time; however there was no significant statistical difference between the two treatment groups with a P-value = 0.478 (Figure 2B)

**Figure 2A:**
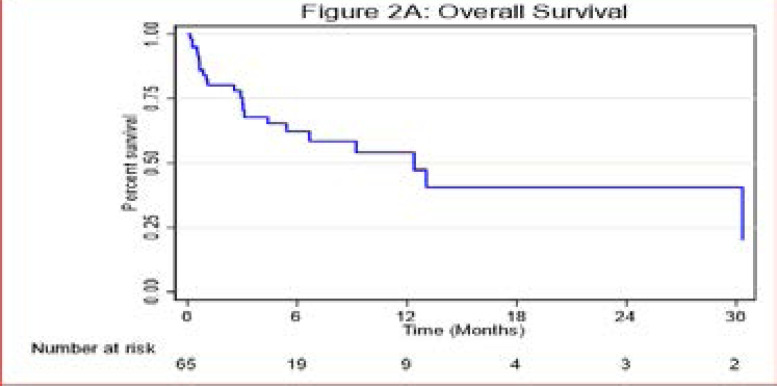
Overall Survival

**Figure 2B:**
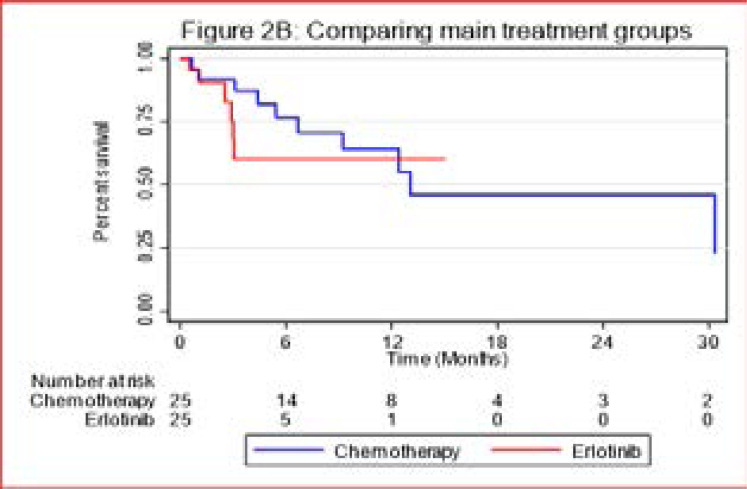
Comparing main treatment groups

In this cohort, patients received an average of 9 cycles (range; 1–12 cycles) of platinum-based chemotherapy as compared to 3 cycles (range; 1 – 6 cycles) of erlotinib. On multivariate analysis using cox proportional hazards regression, poor performance status (ECOG PS 3–4) was associated with worse outcomes (P-value <0.001) in both the crude and adjusted models. Other variables including gender, age, HIV infection, number of sites with metastases, treatment type and histology subtype were not prognostic of adverse outcomes (Table 3)

**Table 3:** Bivariate and multivariable cox regression analysis

Characteristic	cHR (95% CI)	P-value	aHR (95% CI)	P-value
**Sex**				
Male	Ref			
Female	1.3 (0.6, 3.0)	0.518	0.5 (0.1, 4.2)	0.528
**Age (Years)**				
Below 45	Ref			
≥ 45	1.2 (0.4, 3.5)	0.751	4.8 (0.2, 128.7)	0.348
**HIV Status**				
Negative	Ref			
Positive	2.3 (0.8, 6.2)	0.101	1.3 (0.1, 11.7)	0.801
**Sites of metastases**				
1	Ref			
2	0.8 (03, 2.1)	0.635	0.5 (0.1, 3.8)	0.48
3	0.4 (0.1, 3.4)	0.421	4.4 (0.2, 80.8)	0.315
**Treatment type**				
Platinum-based chemotherapy	Ref			
Erlotinib	1.5 (0.5, 4.3)	0.480		
**Performance status**				
1–2	Ref			
3–4	8.1 (2.7, 24.1)	**<0.001**	53.1 (5.0, 569.8)	**0.001**
**Histology**				
Other	Ref			
Adenocarcinoma	1.3 (0.5, 3.3)	0.619	1.1 (0.1, 8.6)	0.986

NB: cHR = Crude hazard ratio, aHR = adjusted hazard ratio

## Discussion

Our findings demonstrate a high burden of late-stage lung cancer at diagnosis in Uganda. We observed that the lung cancer cohort in Uganda constitutes largely of patients that were never-smokers, frequently had histological subtype as adenocarcinoma, and slight disease predominance among females. The clinicopathological profile of Ugandan patient's with non-small cell lung cancer differs considerably from cohorts in other African countries where lung cancer has been reported to occur mainly in males, and in association with tobacco smoking^6^. A recent review of 1,031 patients treated over 5.5 years from a single institution in South Africa showed male predominance, and a relatively high proportion with squamous cell carcinoma^7^.

The median age at diagnosis was 56 years, and 16.2% of patients were below the age of 45 years. This represents an aggressive form of the disease affecting a relatively younger population. The age at diagnosis in the Ugandan cohort is comparable to recent findings from Middle-East and North Africa community where the median age at diagnosis was 56.9 year (±9.5 years)^8^; though much lower than a cohort in South Africa where the median age at diagnosis was 61.6 years^7^. This could be attributed to differences in life-expectancy between the two countries, as well as possible differences in etiopathogenesis. Our youngest patient was 29 years, suggesting the possibility of genetic susceptibility.

The majority of patients had an advanced stage at diagnosis; this has been reported in several centres in Africa, and is attributed to late presentation, limited access to diagnostic modalities, low socioeconomic status, and low literacy levels. This study did not explore the reasons for late presentation. A similar trend has been reported in South Africa, with 81% of patients presenting with advanced disease (stage IIIB/IV) lung cancer at diagnosis^7^. The HIV prevalence in this cohort was higher than the national prevalence of HIV in Uganda; supporting the association between the increased risk of lung cancer in HIV infected patients^6^,^9^. Notably, our cohort were largely young patients and never-smokers, which supports the hypothesis of non-smoking-mediated triggers in this population such as direct effects of HIV on immunosuppression, inflammatory processes, and oncogenic viruses.

Several guidelines recommend a multidisciplinary approach to patients with advanced non-small cell lung cancer^10^. Treatment is tailored to several factors including age, comorbidities, performance status, patient preferences, benefit vs. risks, etc. We found performance status (PS) as the only prognostic factor (p-value < 0.001), which underlines the importance of thorough clinical evaluation prior decision on oncologic treatment. Several studies have demonstrated the prognostic relevance of performance status^11^. However, a poor performance status is likely to correlate with multiple comorbidities, a high smoking exposure, and advanced disease stage^12^.

The use of erlotinib or other tyrosine kinase inhibitors in advanced non-small cell lung cancer is recommended for EGFR-mutated subtypes(10), and is associated with improved survival. The rate of EGFR testing was 8.1% which remains low as compared to reports from other parts of Africa^13^,^14^. Despite the available evidence that EGFR TKI agents are associated with improved survival and quality of life among patients with EGFR-mutated NSCLC, access and cost of these medicines remain a key bottleneck. Also, EGFR mutation typing for patients with advanced NSCLC is not routine practice due to the high cost of this investigation.

Radiotherapy is an essential aspect of cancer treatment either alone or in combination with surgery and chemotherapy^15^. Particularly in Uganda, the advanced stage at diagnosis should translate into a higher radiotherapy utilization rate for palliation of symptoms and pain management. In this cohort, access to radiotherapy was extremely low. This was probably due to radiotherapy access constraints in Uganda at the time of the study.

There were several limitations to this study. First, it was a retrospective study, and hence some aspects of care including access to palliative care services, symptom control, and quality of life could not be measured. Secondly, patients had imaging done from several facilities and these records were not fully accessible to assess response to treatment. Thirdly, there was a high burden of out of pocket expenditure on cancer treatment for patients during the study period, barring compliance to treatment procedures.

## Conclusion

Patients with non-small cell lung cancer in Uganda frequently presented with late-stage disease at diagnosis. The majority of patients were female, never-smokers, and had adenocarcinoma histological subtype.

Late-stage at presentation directly contributes to poor outcomes however, there is a need to improve the approach to care for patients with lung cancer in Uganda through establishing an early diagnosis, proper staging workup, aggressive treatment of early/curable cases through a multidisciplinary approach, rational management of advanced cases by palliative intent and promoting scientific research and availability of clinical trials for lung cancer patients in Uganda.
